# Histopathological, biomechanical, and behavioral pain findings of Achilles tendinopathy using an animal model of overuse injury

**DOI:** 10.14814/phy2.12265

**Published:** 2015-01-19

**Authors:** Leila Jafari, Pascal Vachon, Francis Beaudry, Eve Langelier

**Affiliations:** Département de génie mécanique, Université de Sherbrooke, Sherbrooke, Québec, Canada; Département de Biomédecine Vétérinaire, Faculté de Médecine Vétérinaire, Université de Montréal, Saint‐Hyacinthe, Québec, Canada

**Keywords:** Air‐puff stimulation, Bonar score, calcitonin gene‐related peptide, dynorphin A, instrumented plier, mechanical properties, Randall–Selitto test, substance P, treadmill

## Abstract

Animal models of forced running are used to study overuse tendinopathy, a common health problem for which clear evidence for effective and accessible treatments is still lacking. In these models, pain evaluation is necessary to better understand the disease, help design and evaluate therapies, and ensure humane treatment of the animals. Therefore, the main objective of this study was to evaluate pain and pathologic findings in an animal model of moderate Achilles tendinopathy induced by treadmill running. Air puffs, instead of electrical shocks, were used to stimulate running so that pain associated with stimulation would be avoided. Pressure pain sensitivity was evaluated in vivo using a new instrumented plier, whereas spinal cord peptides were analyzed ex vivo with high‐performance liquid chromatography tandem mass spectrometry. Tendon histologic slides were semiquantitatively evaluated, using the Bonar score technique and biomechanical properties, using the traction test. After 8 weeks of treadmill running (2 weeks for adaptation and 6 weeks for the lesion protocol), the protocol was stopped because the air puffs became ineffective to stimulate running. We, nevertheless, observed some histologic changes characteristic of overuse tendinopathy as well as decreased mechanical properties, increased Substance P and dynorphin A peptides but without pressure pain sensitivity. These results suggest that air‐puffs stimulation is sufficient to induce an early stage tendinopathy to study new therapeutic drugs without inducing unnecessary pain. They also indicate that pain‐associated peptides could be related with movement evoked pain and with the sharp breakdown of the running performance.

## Abstract (precis)

The main objective of this study was to correlate pain and pathologic findings in an animal model of moderate Achilles tendinopathy induced by treadmill running. We observed some histologic changes characteristic of overuse tendinopathy as well as decreased mechanical properties, increased Substance P and dynorphin A peptides but without pressure pain sensitivity.

## Introduction

Tendinopathy is a common health problem. Nearly 8% of the population and 42% of middle and long‐distance runners experience Achilles tendinopathy before the age of 45 (Kujala et al. 2005). The consequences of this medical problem include pain, disability, early retirement from sport and work, mental distress, and health‐care costs. Despite the high prevalence of tendinopathy, the pathophysiology of this condition is not fully understood (Rees 2006; Riley 2008) and clear evidence for effective and accessible treatments is still lacking (Skjong et al. 2012). Questions also remain about the occurrence of pain and its correlation with tissue degeneration and cellular modifications.

As human pathologic tissues are nearly unavailable at early stage of injury and since the tendinopathy development is not controlled, in vivo animal models along with ex vivo mechanobiological studies are needed to investigate tendinopathy and improve healing and prevention strategies. While ex vivo studies allow more controlled conditions than in vivo studies (e.g., applied load) they also provide a less realistic biological environment (e.g., blood flow, inflammation, pain), and therefore, both are complementary.

In vivo models for tendinopathy include chemical models (collagenase, cytokines, prostaglandins, and fluoroquinolone injections) as well as mechanical models (electrical muscle stimulation, uphill and downhill treadmill running, fatigue loading, and disuse), which are well reviewed by Dirks and Warden (2011) and Lui et al. (2011). There are no ideal models and each has its advantages and limitations (Dirks and Warden 2011; Lui et al. 2011). Treadmill running can be used to simulate overuse tendinopathy. For example, previous experiments in supraspinatus (downhill running) (Soslowsky et al. 2000) and Achilles (uphill running) (Glazebrook et al. 2008) tendinopathy models showed modified cell and nucleus shapes, collagen fiber disorganization and increased cellularity. However, in other studies, reproducibility was low for the Achilles tendinopathy. This may be explained by using rats of different strains and ages as well as differences in running protocols and evaluation time points (c.f. Table 1). Mechanically induced models are better designed to induce a tendinopathy that reflects the human condition. However, in these models, the process is usually sped up and they may not entirely reflect human chronic tendinopathy (Shepherd and Screen 2013).

**Table 1.** Treadmill running models for the study of tendinopathy in the rat.

Reference	Rat strain	Age; Weight	Adaptation to the treadmill	Running protocol		Results; Time point
				Speed; inclination	Frequency	
Abraham et al. (2011)	Sprague–Dawley (Male)	4 months (~17 weeks); 448.7 ± 10 g	2 weeks; Increased exposition to the treadmill	16.7 m/min; 10° uphill	1 h/day; 5 or 7 days/week	Tendinopathy; Observable differences in histology; 12 weeks
Dirks et al. (2013)	HCR* (Male)	24.8 ± 3.2 weeks; 374.0 ± 30.3 g	2 weeks; Increase in the running duration (5–60 min) and speed (10–25 m/min)	20–30 m/min; 15° uphill	1 h/day; 5 days/week	No tendinopathy; No significant differences in histology; 7 weeks
Glazebrook et al. (2008)	Sprague–Dawley (Male†)	61–63 days (~9 weeks); 300–325 g	4 days; Increase in the running duration (15 to 60 min)	17 m/min; 10° uphill	1 h/day; 5 days/week	Tendinopathy; Significant differences in histology (nuclei density, collagen organization and staining); 12 weeks
Heinemeier et al. (2012)	Sprague–Dawley (Male)	12–13 weeks†; 363 ± 4 g	1 week; Increase in the running duration (10 to 60 min)	17–20 m/min; 10° uphill	1 h/day; 5 days/week	Tendon improvement; Significant differences in biomechanics (failure stress/body weight and modulus); 12 weeks
Ng et al. (2011)	Sprague–Dawley (Female)	3 months (~13 weeks); 292–360 g	1 week; Increase of the running duration (20–60 min) and speed (10– 17 m/min)	17 m/min; 20° downhill (Running on hind legs)	1 h/day; 7 days/week	Tendinopathy; Observable differences in histology Significant differences in biomechanics (strength and stiffness); 7 weeks
Scott et al. (2007)	Sprague–Dawley (Male)	Not mentioned	2 weeks; Increased exposition to the treadmill	16.7 m/min; 11° downhill (Shoulder tendinopathy)	1 h/day including 5 min pause after 8 weeks; 5 or 7 days/week	No tendinopathy; No significant differences in histology; 4 and 8 weeks; Shoulder tendinopathy; Significant differences in histology (global Bonar score and proliferation); 12 and 16 weeks
Silva et al. (2011)	Wistar (Male)	11–12 weeks; 220–250 grams	2 weeks; 80 min at 13.4 m/min	26.8 m/min; 10° uphill	1 h 20 min/day including 10 min warm‐up and 10 min cool‐down 5 days/week	Tendinopathy; Significant differences in histology (cellularity, microtearing, collagen deposition and GAG); 4, 8, and 16 weeks
Soslowsky et al. (2000)	Sprague–Dawley (Male)	521 ± 33 g at death	Not mentioned	17 m/min; 10° downhill (Shoulder tendinopathy)	1 h/day; 5 days/week	Shoulder tendinopathy; Observable differences in histology (cellularity, cell shape and collagen organization) Significant differences in biomechanics (maximum stress and tissue modulus); 4, 8, and 16 weeks
Yoshida et al. (2014)	Wistar (Male)	16–18 weeks; 350–400 g	2 weeks; Increase in the running duration (5–60 min) and speed (10–25 m/min)	0.5 h m/h (8.33 m/min; 10% inclination (Patellar and rotator cuff &#6tentendinopathies)	40 km; 40 hours	Patellar insertion tendinopathy; Observable differences in histology (microtears and lamination); 28 days after the 40‐day running protocol; Less tendinopathy‐specific histopathological change for the rotator cuff at insertion site.

† Approximated using Charles‐River weight chart.

* HCR rats were selectively bred for high‐capacity running (Dirks et al. 2013).

In these in vivo tendinopathy animal models, one should evaluate pain, as highlighted by Lui et al. (2011) in a recent review of existing models. This information would deepen our understanding of the disease, help design and evaluate therapies, and ensure humane treatment of the animals.

It is recognized that tendon degeneration leading to progressive tendinopathy can be associated with pain (Sharma and Maffulli 2006). It has been suggested that high concentrations of lactate in the Achilles tendon may be responsible for this pain (Alfredson et al. 1999, 2002). Substance P (SP), a well‐characterized pain‐related peptide, and opioids have been identified as biomarkers found in collagenous tissues of Achilles tendinopathy (Ackermann et al. 1999). Consequently, peripheral sensitization mechanisms explain pain originating from sensory nerves found in tendons.

Peripheral tissue injury leads to central neurogenic changes affecting nociceptive signal transmission and modulation. SP and calcitonin gene‐related peptide (CGRP) are two nociceptive peptides found in the dorsal horn of the spinal cord that increase with chronic pain (Campbell and Meyer 2006). Furthermore, spinal endogenous opioid peptide release is increased in chronic pain states (Lai et al. 2006) and SP interacts with this opioid endogenous system (Zubrzycka and Janecka 2000). One of these endogenous peptides, dynorphin A (Dyn A), is increased in chronic articular pain (Ferland et al. 2011). There are therefore certain peptides that can be evaluated as biomarkers of tendinopathic pain. Understanding the pain mechanisms in chronic tendinopathy is essential to develop treatments. For years, tendinopathy has been treated using classical treatments for acute injuries such as rest, ice and anti‐inflammatory medications, even though the histopathology of the effect tendons shows no inflammation (Almekinders and Banes 2007).

However, to study the biomarkers of tendinopathic pain in a treadmill running models, a nonpainful incentive must be used instead of electrical shocks to stimulate running by avoidance. Moreover, since treadmill protocols are time consuming and since biomarkers analyses are conducted on tissues from euthanized animals, a new tool to assess pressure pain threshold in the Achilles tendon in vivo is desirable.

Therefore, the objectives of this study were as follows: (1) to assess different aspects of the rat model of Achilles tendinopathy induced by treadmill running (running behavior, knee and tendon pathology, end of study central nervous system pain‐related peptides), (2) to validate if air puffs can be used to elicit running instead of electrical shocks in the study of tendinopathy associated pain, and (3) to assess the potential use of a new instrumented plier to evaluate pressure pain sensitivity in the affected Achilles tendon. In this study, we subjected rats to an uphill running protocol of several weeks. Histologic and biomechanical analyses of the Achilles tendons were performed to confirm the tendinopathy. We evaluated pain levels using a new instrumented plier for pressure pain sensitivity as well as concentration of SP, CGRP, and Dyn A in the spinal cords. Pain analysis following an injection of collagenase in the Achilles tendon was conducted in a preliminary study to validate the new instrumented plier in a tendinitis model.

## Materials and Methods

### Ethical approval

The Animal Care and Use Committee of the Faculty of Veterinary Medicine approved the protocol prior to animal experimentation. All procedures are in accordance with the guidelines of the Canadian Council on Animal Care.

### Animals

Sixteen Sprague–Dawley rats (12–16 weeks, BW 470–540 g at the beginning of the protocol) were used for the treadmill study (eight runners; eight nonrunners). Five rats (7–8 weeks; BW 250–275 g) were used for the collagenase injection study. They were housed in a standard research environment (temperature: 21 ± 1°C; fresh filtered air (15 changes/h); humidity 40–60%; light–dark cycle (12 h:12 h)). Rats were pair‐housed in polycarbonate cages (Ancare, Bellmore, NY) with hardwood bedding (Beta chip; North‐Eastern Products, Warrensburg, NY) and acclimated for 7 days prior to the study. The animals were fed a rodent chow (Charles River Rodent Chow 5075, St‐Constant, Québec, Canada) and received tap water, both ad libitum.

### Treadmill study

#### Treadmill Design

**Figure 1.**
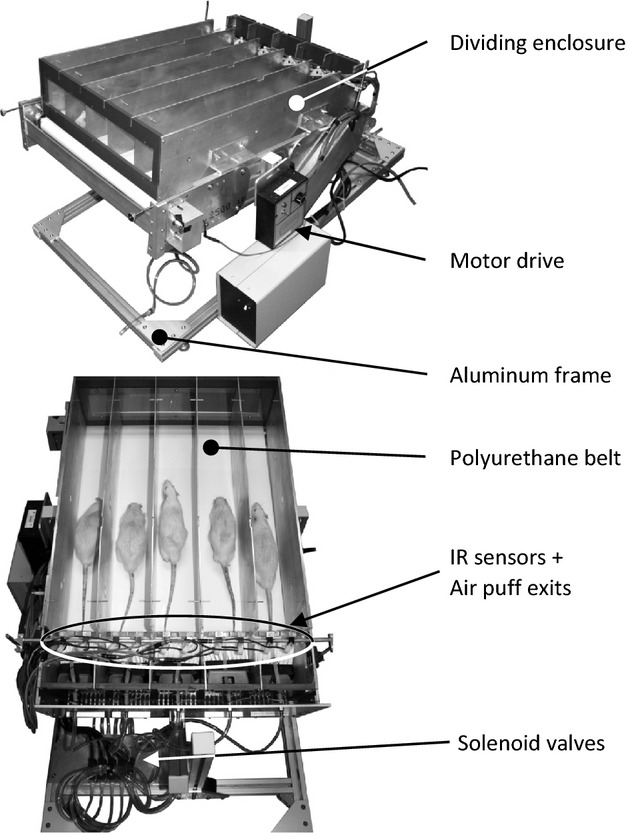
Custom treadmill. Five rats run on a polyurethane belt in separate corridors. To stimulate running, resting rats are detected by infrared sensors which trigger the injection of air puffs via the opening of solenoid valves connected to a cylinder of compressed air.

The treadmill consists of a polyurethane belt (JCM245WU, Nuera Industriel, Laval, Canada) driven by a 1/8HP dc motor (CM31D18NZ10 and Speedmaster, Leeson, Grafton) and is supported by a frame made of aluminum extrusions (Fig. 1). Over the treadmill, an enclosure divides the running space into five single running corridors. At the tail end of the treadmill, infrared sensors (GP2D120XJ00F, Sharp, Osaka, Japan) detect the presence of resting animals and trigger the opening of solenoid valves (14.5137; Airmax Industriex, Granby, Canada) connected to a cylinder of compressed air. Air puffs stimulated running by avoidance. This was required since rats naturally dislike this nonpainful stimulus. Otherwise, they would rather rest at the back of the treadmill on a small platform which was designed to prevent the animal's foot to jam between the belt and the enclosure as well as to evacuate excrements. The sensor acquisition and processing as well as the valve control are carried out with an acquisition card (National Instruments, Austin, TX) and custom software (Labview, National Instruments).

#### Running protocol

Following arrival and a 1‐week acclimation, 16 rats were trained for 2 weeks by daily increasing the distance (25–1065 m/day), speed (5 to 13.4 m/min), and inclination (0 to 10°). The rats were then divided in two groups (*n* = 8 runners and *n* = 8 controls) based on their willingness to run (the lower the number of air puffs, the higher the willingness to run). The runners were subjected to a 6‐week running protocol at a 10° inclination to induce eccentric muscle contractions and increase the risk for tendon injury (Glazebrook et al. 2008). Each day, their running session began with a 10‐min warm‐up at increasing speed and ended with a 10‐min cool‐down at decreasing speed. In between, they ran 60 min at their maximal capacity (~17.75 m/min to 18.55 m/min) which was evaluated using the number of air puffs. The objective was to increase the treadmill speed as much as possible, within the limits of humane treatment. During the running period, the animals were monitored for paw irritation, limping and excessive number of air puffs. When needed, animals were removed temporarily (specified below) from the running protocol to heal. Animals were weighed every week. At the end of the running protocol, the rats were killed using carbon dioxide.

#### Evaluation of pressure pain sensitivity on live animal

**Figure 2.**
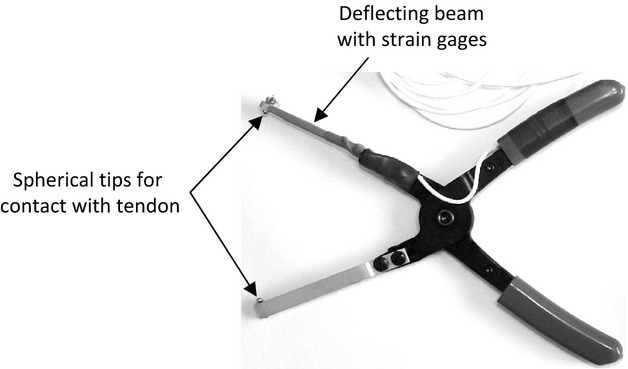
Instrumented plier for the evaluation of pressure pain sensitivity in the Achilles tendon in vivo. The pressure on the rat tendon induces the bending of the measuring beam. The resulting strain is measured with two strain gages in half bridge.

An instrumented plier (Fig. 2) was designed to evaluate pressure pain sensitivity in the tendon, which is the clinical equivalent of the pinch test. The instrumented plier is inspired by the Randall–Selitto test (Randall and Selitto 1957) in which an increasing mechanical force is applied on the paw of the animal. The maximal force reached when the animal voluntarily withdraws its paw is recorded. The instrument plier is used in a similar way. Briefly, two strain gages (062UW, Vishay Precision Group, Malvern) were applied on a beam in half bridge to measure its strain under load, that is, when pressing on the rat Achilles tendon.

Before euthanasia, each rat was constrained until its hind leg was relaxed. An increasing force was then applied on its Achilles tendon until the animal withdrawal reflex. The maximum force was recorded. The procedure was repeated on the other Achilles tendon. Animals were habituated to confinement and hind leg manipulations a few days before pressure pain sensitivity testing.

#### Histologic preparation and evaluation of Achilles tendons

**Figure 3.**
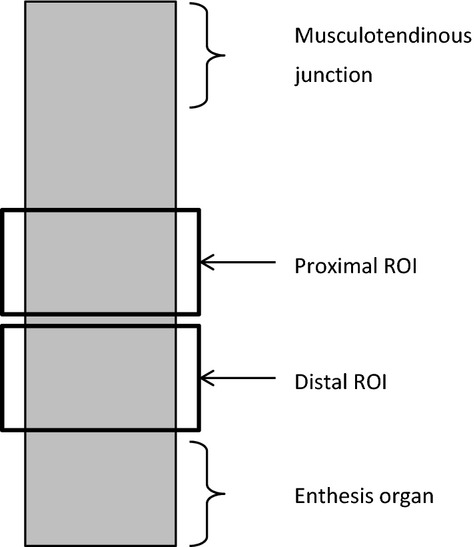
Schematic representation of a longitudinal tendon section showing the position of the two ROIs for histologic analysis.

Following euthanasia, Achilles tendons from one hind leg were harvested for histology. (The Achilles tendons from the other hind leg were used for biomechanical evaluation). They were first fixed in 10% neutral‐buffered formalin for at least 24 h and then embedded in paraffin. Longitudinal sections 5 *μ*m thick were either processed in hematoxylin and eosin stain (HE) or alcian blue stain at pH 2.5 and observed under bright field (HE and alcian blue) and polarized light microscopy (HE). On each sections, two regions of interest (ROIs) were captured (Fig. 3) as preliminary wide‐field observations confirmed that damages were located in the distal portion of the tendon, such as observed in the human Achilles tendon (Józsa and Kannus 1997). Moreover, the enthesis organ was avoided because of its complexity (Benjamin and McGonagle 2009).

For histologic analysis, the images were printed and randomly numbered for blind analysis by two independent observers. Tissue quality was score semiquantitatively between 0 and 3 (Table 2) using the Bonar score technique (Cook et al. 2004). If there was a difference of more than one degree between two scores, both observers scored the images again together. The most outlying score was then replaced with this new score. Then, for each image, an average of the two scores was calculated. The global score was calculated as the average score of all criteria.

**Table 2.** Boundary grades used for the semiquantitative scoring of histologic images of tendons.

Criteria	Grade 0	Grade 3
Nucleus morphology (HE)	Thin and elongated	Large and round
Cytoplasm morphology (HE)	No obvious cytoplasm	Abundant cytoplasm
Cellular arrangement (HE)	Regular cell density	Presence of cell clusters
Collagen arrangement (HE under polarized light)	Parallel arrangement	Multi‐directional arrangement
Vascularity (HE)	0 blood vessels per ROI	≥5 blood vessels per ROI
Ground substance (Alcian blue)	No stainable ground substance	Abundant ground substance

Tenocyte density was evaluated using a grid over the images. The number of cells per square was counted avoiding the regions with blood vessels because the high density of endothelial cells would distort the results.

#### Histologic preparation and evaluation of knee joints

Sprague–Dawley rats are predisposed to osteoarthritis with aging. As osteoarthritis could induce pain and therefore modify the spinal cord peptide analysis, we examined if treadmill running induced knee cartilage degradation.

Following euthanasia, the knee joints were dissected free of muscle and fixed in a 10% buffered formaldehyde solution for 48 h and transferred into a decalcification buffer (pH 7.3) containing 20% ethylenediaminetetraacetic acid (EDTA) for 14 days. Afterward, joints were rinsed with tap water and placed in 10% buffered formaldehyde solution for a maximum of 48 h prior to paraffin embedding. Coronal sectioning was performed and 4 *μ*m paraffin sections were stained with safranin O‐fast green to assess articular cartilage changes. Sections were mounted and observed by two pathologists (Christiane Girard DVM, DACVP and Pierre Hélie DVM, DACVP; both from the department of veterinary pathology of the Université de Montréal). The knee histology was evaluated with the following criteria: the synovial membrane hypertrophy (0: normal, 1: light, 2: moderate), hyperplasia of synoviocytes (0: absence, 1: two layers, 2: 3 and more layers), stromal granular inflammation (0: absence, 1: presence, cartilage structural changes (0: normal, 1: erosion, 2: ulceration of the hyaline cartilage, 3: ulceration of the calcified cartilage), loss of staining of the femoral condyle or at the tibia plateau (0: uniform staining throughout articular cartilage, 1: 1⁄3 loss of staining in superficial zone of hyaline cartilage, 2: 2/3 loss of staining of hyaline cartilage, 3: loss of staining in all the hyaline cartilage, 4: loss of hyaline cartilage), and cluster formations (0: absence, 1: presence). As there was no sign of cartilage deterioration or loss of proteoglycans when comparing runners and controls, no further analysis was conducted.

#### Biomechanical evaluation of Achilles tendons

Following euthanasia, Achilles tendons from the other leg were harvested for biomechanical evaluation. The proximal fibrous sheath and the calcaneus were kept for specimen mounting in the traction machine (5540; Instron, Norwood, MA). The fibrous sheath was wound around a small triangular file. Both, the sheath and the calcaneus were then fixed between two sand paper strips in the grips. The length of the tendon was measured using a vernier caliper between the centre of the file and the enthesis. The traction test was conducted at room temperature and the tendon was kept moist with PBS during the whole procedure. The tendon was stretch at a speed of 2 mm/min. Load and displacement were recorded at a sampling rate of 100 Hz. On the load–displacement curve, the ultimate tensile force corresponded to the maximum force reached during the test, while the rigidity corresponded to the steepest linear slope. The calculation was performed using equation 1.



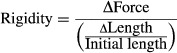



#### Spinal cord peptide analysis

The analytical method used was based on method previously published (Ferland et al. 2011). Lumbar spinal cord segments of all rats were analyzed by high‐performance liquid chromatography–tandem mass spectrometry (HPLC‐MS/MS) using an isotopic dilution technique for the determination of SP, CGRP, and Dyn A concentrations. Following collection, spinal cord samples were immediately snap‐frozen in cold hexane (≈−60°C) and stored at −80°C pending analysis. The tissues were weighed frozen and homogenized following the addition of 0.25% trifluoroacetic acid solution with a ratio of 1:5 (w:v). The samples were sonicated for 20 min and 150 *μ*L of the homogenate were mixed with 150 *μ*L of acetonitrile to precipitate high molecular weight proteins. The samples were vortexed and centrifuged at 12,000 g for 10 min and 150 *μ*L of the supernatant were transferred into an injection vial and spiked with 150 *μ*L of the internal standard solution (deuterium labeled peptides). The vials were capped and vortexed vigorously and 2 *μ*L were chromatographed. The chromatography was achieved using a gradient mobile phase along with a microbore column Thermo Biobasic C8 100 × 1 mm, with a particle size of 5 *μ*m. The initial mobile phase condition consisted of acetonitrile and water (both fortified with 0.1% of formic acid) at a ratio of 5:95. From 0 to 1 min, the ratio was maintained at 5:95. From 1 to 12 min, a linear gradient was applied up to a ratio of 60:40 and maintained for 3 min. The mobile phase composition ratio was reverted at the initial conditions and the column was allowed to re‐equilibrate for 15 min for a total run time of 30 min. The flow rate was fixed at 75 *μ*L/min. The mass spectrometer (Thermo LTQ‐XL) was coupled with the HPLC system using a pneumatically assisted electrospray ion source (ESI). The sheath gas was set to 10 units and the ESI electrode was set to 4000 V in positive mode. The capillary temperature was set at 300°C and the capillary voltage to 45 V. All scan events were acquired with a 100 msec maximum injection time. The mass spectrometer operated for quantitative analyses in multiple reaction monitoring (MRM) mode. Peptide spinal cord concentrations could be associated with mechanical tendon compression using the instrumented pliers. Therefore, we examined differences between groups instead of baseline peptide levels.

#### Collagenase injection study

This pilot study was conducted to evaluate the effectiveness of the pain pressure sensitivity test using the modified pliers. Rats (*n* = 5; BW 250–275 g) were anesthetized with an intraperitoneal injection of a ketamine and xylazine (75 mg/kg and 10 mg/kg, respectively). Right Achilles tendons were injected near the osteotendinous junction with 30 *μ*L of crude collagenase (10 mg/mL saline; Sigma, St‐Louis, MO) using a 30G needle. Live animals were evaluated for tendon sensitivity (pressure pain sensitivity of both right and left legs) 24 and 72 h following the collagenase injection. As for the tendinopathy study, selected spinal cord peptides were analyzed after euthanasia.

#### Statistical analysis

In the treadmill and collagenase injection studies, Bonar scores as well as biomechanical and peptide results were compared using the nonparametric Mann–Whitney test, whereas the pain sensitivities were compared using the Wilcoxon matched‐pairs signed rank test. Outliers were detected using the ROUT method developed for Prism 6 (GraphPad Software Inc, La Jolla, CA) with *Q* = 2%. They were removed from the analysis.

## Results

### Progression of the running experiment

**Figure 4.**
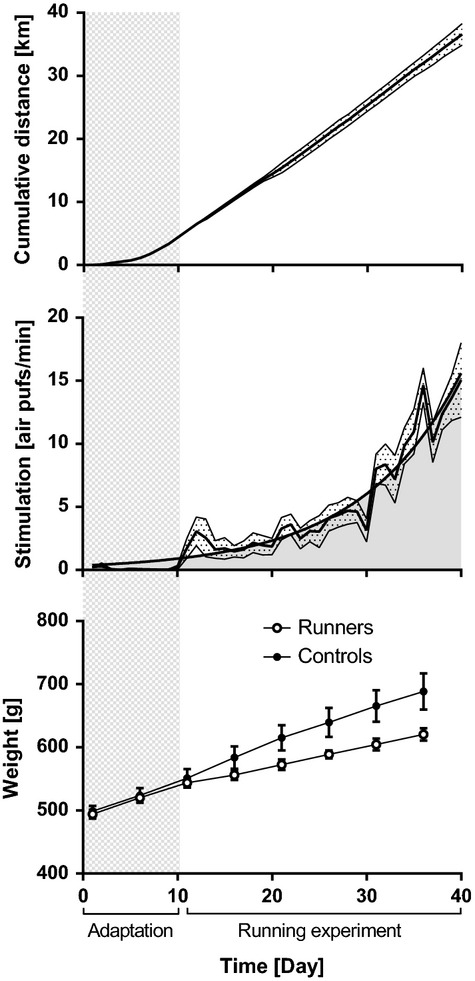
Progression of the running experiment including 2 weeks of adaptation and 6 weeks of running. Rats ran, on the average, a total of 36.6 km. The experiment was stopped because the stimulation became too high. The number of air puffs followed an exponential curve and reached 15 air puffs/min (900 air puffs/h). Data are shown as mean ± SEM.

Originally, the running experiment was supposed to be longer than 6 weeks. However, the rats did not respond as well to the air puffs as time went by (Fig. 4) and were less stimulated to run. We therefore decided to end the running protocol. During this period (and the 2 weeks for adaptation), the rats ran an average of 36.6 km (Fig. 4). The differences in the weight of the runners and controls increased with time (Fig. 4).

During the experiment, we did not observe limping or paw irritation. However, it is worth noting that two rats were removed from the running protocol for 3 days and one rat, for 1 day, in order to heal. These animals caught their claws in the treadmill. The injury and the bleeding changed their willingness to run as well as their gait.

### Histology of the Achilles tendons

**Figure 5.**
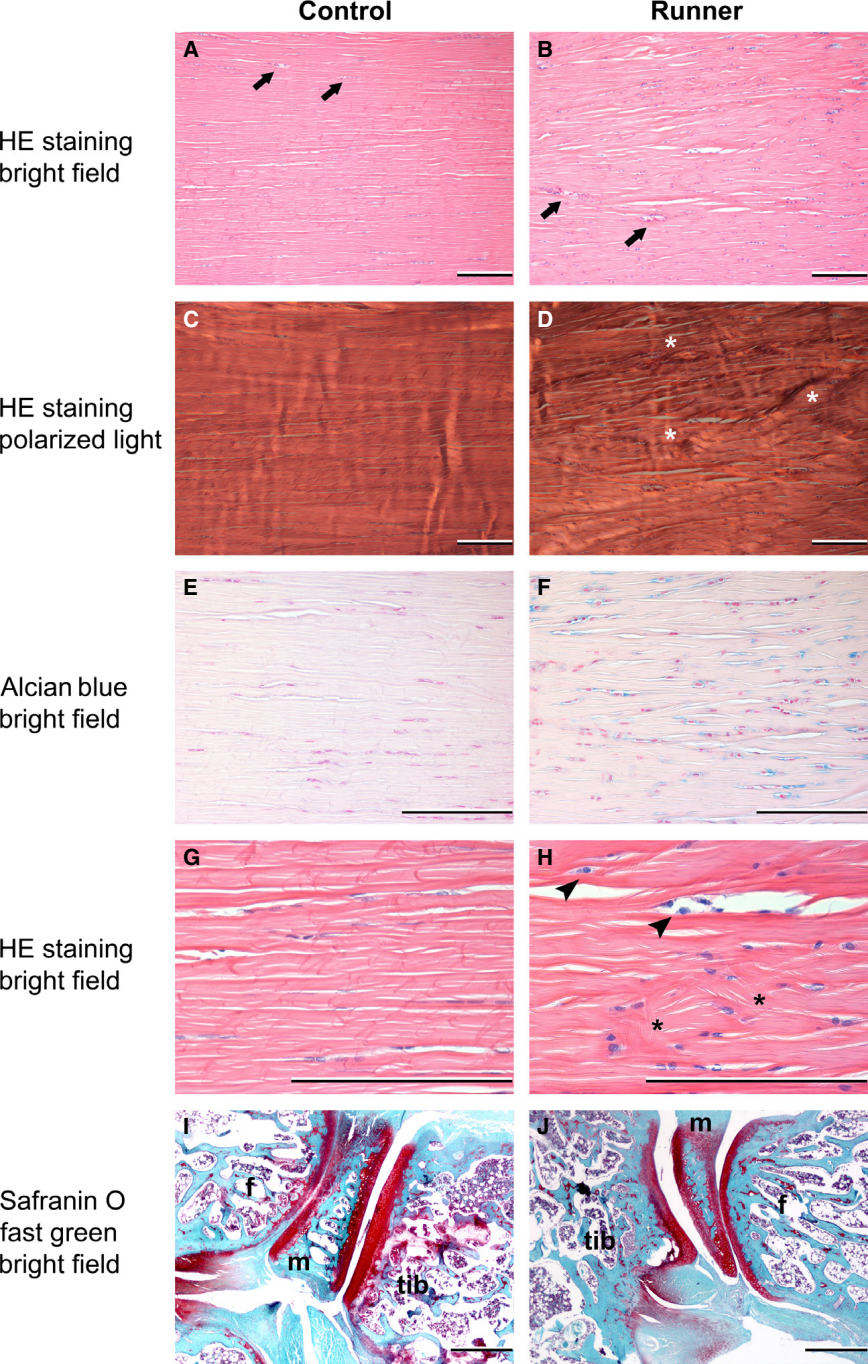
Micrographs of the proximal ROI of Achilles tendons (A–H; Bar = 200 *μ*m) and micrographs of knee sections (I–J; Bar = 500 *μ*m; Tib tibia, m meniscus, f femur) for a control rat and a runner. Arrows indicate blood vessels, asterisks indicate collagen disorganization, and arrow head indicate round nucleus and abundant cytoplasm. In the example shown, the Bonar score for the runner tendon is: Nucleus morphology: 3, Cytoplasm morphology: 2, Cellular arrangement: 2, Collagen arrangement: 2, Vascularity: 2, Ground substance: 3, Cell density: 64 and the Bonar score for the control tendon is: Nucleus morphology: 0, Cytoplasm morphology: 1, Cellular arrangement: 0.5, Collagen arrangement: 1.5, Vascularity: 2, Ground substance: 1, Cell density: 39. The scores for the control (I) and runner (J) knee histopathology are zero (details of parameters evaluated found in text). Images were subjected to color balance and brightness/contrast adjustments.

Micrograph examples and related Bonar scores are shown in Fig. 5. Please note that, in order to assure detail visibility for the readers, each micrograph shows a smaller region of the investigated ROI.

**Figure 6.**
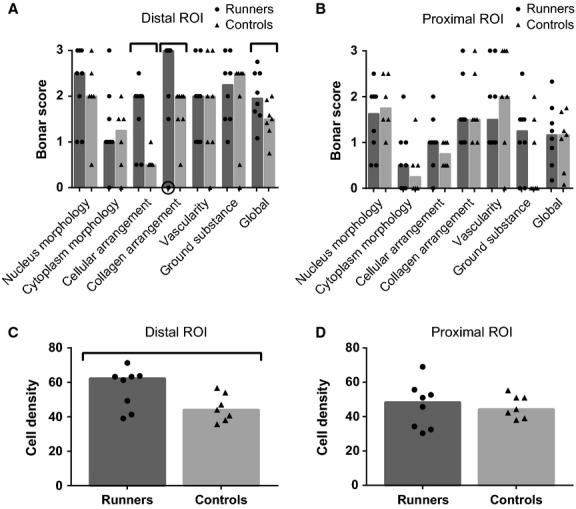
Histologic results for the Achilles tendons of runner and control rats. A–B: Bonar scores. C–D: Cell density. Columns represent the median, and symbols represent data. Encircled symbol represents an outlier removed from the analysis. Horizontal bars stand for significant differences between groups (*P* < 0.05)

Histologic analyses (Fig. 6) revealed a significant difference between the Bonar scores from runner and control groups for cellular arrangement, collagen arrangement and global score in the distal ROI. There was also a significant difference in cellular density in the distal ROI. Based on these results, tendons from runners had a higher cell density, more cell clusters and more disorganized collagen than tendons from controls in the region above the enthesis organ.

### Histology of the knee joints

Microscopic observations of histologic preparations of knee joints revealed no differences between runners and controls (Fig. 5).

### Biomechanics

**Figure 7.**
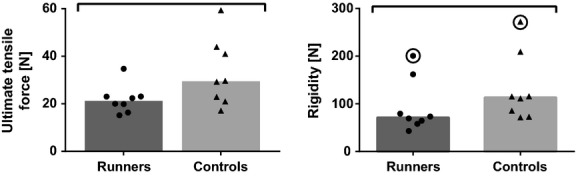
Biomechanical results for the Achilles tendons of runner and control rats. Columns represent the median, and symbols represent data. Encircled symbols represent outliers removed from the analysis. Horizontal bars stand for significant differences between groups (*P* < 0.05).

Tissue rupture occurred in the fibrous sheath at the interface between the file and the sand paper. Based on the measured ultimate tensile force and rigidity (Fig. 7), the tissues from runners were weaker than tissues from controls. For runners, we note a decrease of 37.7% for the median of ultimate tensile force, and a decrease of 28.1% for the median of rigidity.

### Pressure pain sensitivity

**Figure 8.**
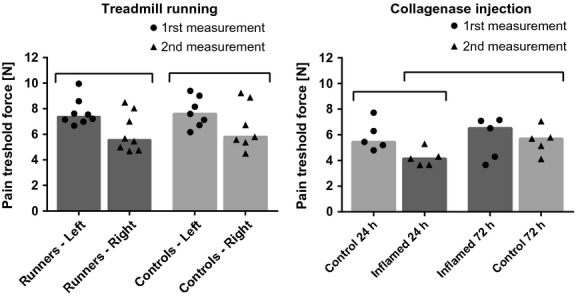
Pain threshold measured with the instrumented plier for the treadmill and collagenase injection studies. Columns represent the median, and symbols represent data. Horizontal bars stand for significant differences between groups (*P* < 0.05).

The pressure pain sensitivity evaluated using the instrumented plier in the treadmill study seems to be affected by the evaluation order. The pain threshold force measured in the first leg was statistically higher than the force measured in the second leg (Fig. 8). However, there was no difference between the forces measured in the first legs of the runners versus controls, nor between the forces measured in the second legs of the runners versus controls.

In the collagenase injection study, to overcome the increased sensitivity between two pain evaluations, we tested the hind legs and compared the pain thresholds as summarized in Table 3.

**Table 3.**
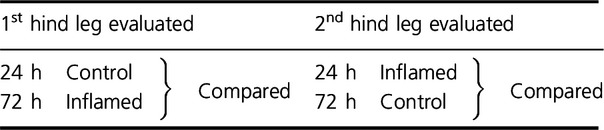
Leg order and threshold comparison for pain evaluation in the collagenase study.

We observe a statistically significant difference between the pain threshold force in the inflamed tendons at 24 h and the control tendons at 72 h (Fig. 8). It is worth noting that both pain thresholds were evaluated in the same order, that is, both were the second measurement, thus eliminating the effect of evaluation order.

### Spinal cord peptide analysis

**Figure 9.**
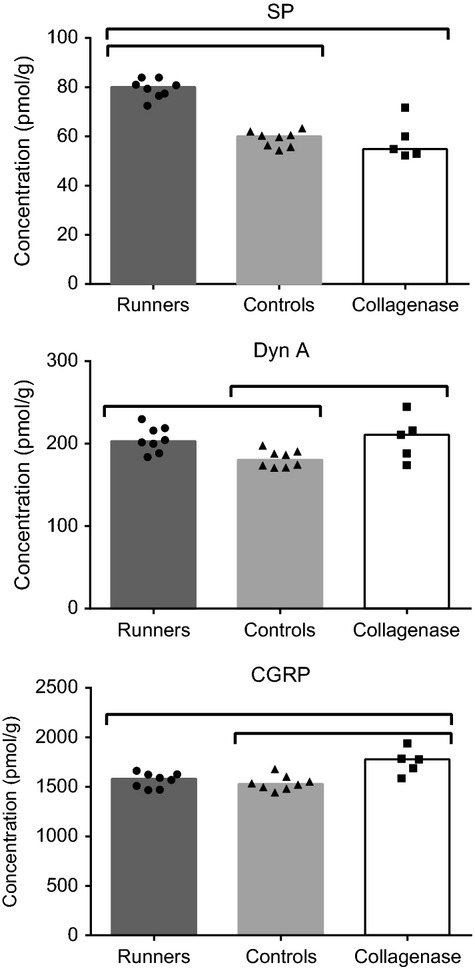
Spinal cord peptide concentrations for the treadmill and collagenase injection studies. Columns represent the median, and symbols represent data. Horizontal bars stand for significant differences between groups (*P* < 0.05).

In the treadmill study, the concentration of SP and Dyn A was significantly higher for runners compared with the control group (Fig. 9). When comparing results from the treadmill and collagenase injection study (Fig. 9), we observe similar concentration of SP in controls and collagenase‐injected animals, an increase in Dyn A concentration in collagenase‐injected animals over controls, as well as an increase in CGRP concentration in collagenase‐injected animals over both controls and runners.

## Discussion

The first and main objective of this treadmill study was to correlate pain and pathologic findings in a moderate Achilles tendinopathy in an animal model based on treadmill running. After 8 weeks of treadmill running (2 weeks for adaptation and 6 weeks for the lesion protocol), we observed some histologic changes characteristic of overuse tendinopathy as well as decreased mechanical properties, increased SP and Dyn A peptides but without pressure pain sensitivity.

### Limitations

The forced treadmill running model shows some limitations as pointed out by Shepherd and Screen (2013). It remains unknown if small animals naturally encounter tendinopathy in real life. Moreover, the etiology of overuse tendinopathy may vary between species. Finally, in this model, the overuse process is sped up and may not represent the exact process of human chronic tendinopathy which can develop over months or years. Lui et al. (2011) also underline that rats and rabbits, although popular animal models for tendinopathy, require carefulness as they may use different mechanisms for balancing, locomotion, and pain perception than human. However, the forced treadmill running model using rats, together with other animal fatigue models, helps to understand the development of tendinopathy.

On the basis of the literature (Table 1), we expected to observe a moderate tendinopathy after 8 weeks. However, we stopped our running protocols after only 6 weeks because the number of air puffs/min, reflective of a poor running performance, reached a point where we judged the experiment ineffective. At this point, many rats would snuggle up at the back of the treadmill on a small resting platform under the IR sensors and received batches of air puffs. Presence of pain combined with a decreased motivation to run may explain these results. Although electrical shocks used in commercial treadmills may appear to be somewhat more effective to stimulate running, the pain and negative reinforcement induced by this approaches is unacceptable in pain studies. We initially chose air puffs to improve the ethical aspect of the protocol and our results suggest that our protocol is adequate to simulate a moderate tendinopathy.

During the running protocol, three rats were allowed to rest between 1 and 3 days in order to heal because they caught their claws in the treadmill. We verified but found no correlation between this rest period and the degree of tendinopathy.

In our study, we encountered unexpected difficulties and results. First, using the instrumented pliers, it was difficult to apply pressure to the Achilles tendon in a stable manner as the extremities of the instrument were spherical. Sometimes, the spheres slipped toward the space between the bone and the tendon. We therefore propose to replace the spheres with cylinders, which would allow adequate contact at the tendon levels. Second, we observed that for two consecutive measurements on the same animal but different legs, the second measurement is lower than the first one, as if the animal adapted its response to the stimulus. This finding agrees with those of Anseloni et al. (2003) and Taiwo et al. (1989) showing a decreased pain threshold to the Randall–Selitto test during training to sensitivity, even within a daily training session (Anseloni et al. 2003). This can be problematic when comparing two different conditions, for example, a tested leg versus a sham or control leg as in our collagenase injection study. However, based on these results, training sessions could help to bypass this drawback however further investigation is required. Moreover, Winter and Flataker (1965) reported that they observed no significant difference between left and right paws in preliminary tests when applying pressure to the rat foot using a piston and a manometer. Unfortunately, their testing protocol does not include many details. Other strategies could be to use the average of multiple consecutive measurements during which legs are alternated or increase the number of measurement time points and alternate the first and second legs. Then, however, one should only compare the first measurements together and the second measurement together. Finally, the bulk of inflammation lasts around 3 days, which may explain why the pressure pain thresholds are not significantly different in collagenase‐injected tendons after 72 h and control tendons after 24 h. More measurements on more animals would have given us a more accurate view of pain progression over that period.

We also faced some challenges during the biomechanical analysis. First, it is well‐known that tendons are difficult to securely fix in a traction machine without inducing stress concentration. This difficulty is increased by the small size of rat Achilles tendons. Therefore, despite our efforts, we observed tissue rupture in the fibrous sheath at the interface between the file and the sand paper. One can imagine tendon properties to be higher than actual findings. Second, the evaluation of tendon cross‐sectional area was problematic for different reasons. The orientation of the three tendon subunits (one from soleus and two from gastrocnemius) can vary during manipulation and change the whole tissue geometry. Moreover, modeling the transversal geometry of the Achilles tendon as a circle or an ellipse could induce large error in the evaluation of cross‐sectional area as observed for tail tendon (Parent et al. 2010). Because we did not have access to an optical micrometer adapted to Achilles tendon, we chose to evaluate forces instead of stresses. This may contribute to the large dispersion of the biomechanical results.

Finally, we do not know the exact orientation of the histologic sections (frontal vs. sagittal). To bypass this problem we did not consider the images in the enthesis organ which has a complex structure (Benjamin and McGonagle 2009). Moreover, as we were able to recognize the soleus and gastrocnemius subunits, we concentrated our work on the central section of the gastrocnemius subunits. Using these strategies, we are confident about our results.

### Development of a tendinopathy

Taken together, our results confirm published findings. Three groups (Glazebrook et al. 2008; Abraham et al. 2011; Silva et al. 2011) using Sprague–Dawley and Wistar rats and uphill running protocols also observed histologic signs of tendinopathy (Table 1) while two groups (Heinemeier et al. 2012; Dirks et al. 2013) using Sprague–Dawley and HCR rats and uphill running protocols did not observed histologic signs of tendinopathy. However, it is worth noting that HCR rats were selectively bred for high‐capacity running, which may explain the different results of Dirks et al. (2013). In an attempt to understand their unexpected results, Heinemeier et al. (2012) highlight the difference in the histologic scoring protocols. They also mention that “it cannot be excluded that the progression from healthy adaptation to maladaptation is a fine balance, and that small, seemingly inconsequent, differences may be important.”

Regarding the tendon mechanical properties, the observed decreased strength and stiffness are consistent with the histologic findings and other studies. Both human and animal studies of tendinopathy show decreased mechanical properties (Soslowsky et al. 2000; Ng et al. 2011; Helland et al. 2013).

### Correlation with pain

Our results show an increase in SP and Dyn A with treadmill running as well as CGRP and Dyn A with collagenase injection. Substance P (Gotoh et al. 1998; Schubert et al. 2005; Lian et al. 2006) and CGRP (Lui et al. 2010) are present in peripheral nerves found in tendons and have been associate with pain‐related tendinopathy. No study has previously looked at modifications in central pain‐related peptides in the spinal cord of animal models of tendinopathy (Liu et al. 2010). In a previous study (Ferland et al. 2011), we found that the concentration of CGRP in the spinal cord rapidly increased following the induction of an osteoarthritic pain in rats, followed by SP, where spinal cord concentrations increased days later. In the treadmill running model, no articular degeneration occurred with exercise and no tendon associated pain was demonstrated using the instrumented plier. However, we only tested one area per tendon and it is possible that other areas could have a lower pressure‐pain threshold. Moreover, pain may only appear through exertion and stop after activity. The pain‐associated peptides could therefore be associated with pain during running or pain in other tissues such as ankle pain (not evaluated). Foot skin irritation does not seem to be associated with pain as tissue irritation was not observed but pain associated with caught claws could be present in three rats. Pain peptides modifications could very well be associated with the sharp breakdown of the running performance seen at the end of the treadmill study. On the other hand, tendon pressure‐induced pain seen with collagenase injection clearly suggests pain associated with the increase of both pain‐related peptides, CGRP and Dyn A. Spinal endogenous opioid peptides, such as dynorphins (Wang et al. 2001), are increased in chronic pain states (Lai et al. 2006). Although an expected analgesic effect is associated with opioids, dynorphins‐injected intrathecally around the spinal cord can induce a chronic pain state (Tan‐No et al. 2002). Therefore, increases of pain‐related peptides occurring with treadmill running could be related to a moderate tendinopathy induced by forced treadmill running. According to literature, the collagenase and tendinopathy models have clearly different pathologic mechanisms. In the collagenase model, there is inflammation and the extent of tendon damage is more important. Pain‐related peptides have been demonstrated in spinal cords in many different animal models of chronic pain. These molecules are known to be responsible for central sensitization dorsal horn cells which transmit pain to supraspinal areas of central nervous system. This pilot study suggests that this could be a route to follow to understand pain in these tendinopathy models.

### Originality

To our knowledge, this is the first study to correlated histology, mechanical properties and pain analyses in an animal model of overuse tendinopathy. The results allow having a more complete view of early stage tendinopathy. The correlation of histology and mechanical properties with pain is necessary to better understand tendinopathy and develop treatments for this disease. Moreover, we would suggest that air puffs are sufficient to induce an early stage tendinopathy to study new therapeutic drugs without inducing unnecessary pain caused by electrical shocks. Finally, preliminary results using the instrumented plier highlight the potential of this new device to assess tendon pain in animal study of tendon pathology such as tendinopathy and arthritis. However, more investigations are required with new extremities for the pliers, more testing areas along the tendon and animal acclimation to improve pain sensitivity results. More animals are needed to increase statistical power.

## Acknowledgments

We thank Micheline Fortin for the preparation of tendon histologic sections.

## Conflict of Interest

None declared.
